# Persistence of the SARS-CoV-2 Antibody Response in Asymptomatic Patients in Correctional Facilities

**DOI:** 10.3389/fmicb.2021.789374

**Published:** 2021-11-10

**Authors:** Xiaodong Tian, Wenguo Jiang, He Zhang, XiXi Lu, Libo Li, Wenjun Liu, Jing Li

**Affiliations:** ^1^CAS Key Laboratory of Pathogenic Microbiology and Immunology, Institute of Microbiology, Chinese Academy of Sciences, Beijing, China; ^2^School of Life Sciences, University of Science and Technology of China, Hefei, China; ^3^Jining Center for Disease Control and Prevention, Shandong, China; ^4^Savaid Medical School, University of the Chinese Academy of Sciences, Beijing, China; ^5^Institute of Microbiology, Center for Biosafety Mega-Science, Chinese Academy of Sciences, Beijing, China

**Keywords:** COVID-19, SARS-CoV-2, antibody response, asymptomatic infection, persistence

## Abstract

SARS-CoV-2 has caused a global health disaster with millions of death worldwide, and the substantial proportion of asymptomatic carriers poses a huge threat to public health. The long-term antibody responses and neutralization activity during natural asymptomatic SARS-CoV-2 infection are unknown. In this study, we used enzyme-linked immunosorbent assays (ELISA) and neutralization assay with purified SARS-CoV-2S and N proteins to study the antibody responses of 156 individuals with natural asymptomatic infection. We found robust antibody responses to SARS-CoV-2 in 156 patients from 6 to 12 months. Although the antibody responses gradually decreased, S-IgG was more stable than N-IgG. S-IgG was still detected in 79% of naturally infected individuals after 12 months of infection. Moderate to potent neutralization activities were also observed in 98.74% of patients 6 months after infection. However, this proportion decreased at 8-month (46.15%) and 10-month (39.11%) after infection, respectively. Only 23.72% of patients displayed potent neutralization activity at 12 months. This study strongly supports the long-term presence of antibodies against SARS-CoV-2 in individuals with natural asymptomatic infection, although the magnitude of the antibody responses started to cripple 6 months after infection.

## Introduction

The pandemic of coronavirus disease 2019 (COVID-19) caused by severe acute respiratory syndrome coronavirus 2 (SARS-CoV-2) has posed a severe threat to global public health and economic development (Galipeau et al., 2020; Gudbjartsson et al., 2020; Li and Liu, 2020). World Health Organization [WHO], 2020 has confirmed more than 173 million COVID-19 cases worldwide, resulting in more than 3.7 million deaths in June 2021. Based on the severity of the disease, COVID-19 has been classified into four severity levels: mild, moderate, severe, and critical (Hashem et al., 2020; Ibarrondo et al., 2020). The proportion of patients with severe or critical diseases may vary with age and health status, but most have mild to moderate disease (Figueiredo-Campos et al., 2020; Wang et al., 2020). However, with the global coronavirus outbreak, there is increasing evidence that many patients with COVID-19 are asymptomatic but can transmit the virus to others (Garrido et al., 2021; Zheng et al., 2021).

The induction of effective early immune control of SARS-CoV-2 and a durable immune response is critical to prevent severe disease and defend against re-exposure (Anand et al., 2021; Cohen et al., 2021; Garcia-Beltran et al., 2021). Many published studies have explored the magnitude and longevity of the COVID-19 antibody response. To our knowledge, the observation period for most studies on SARS-CoV-2-specific antibodies was 8 months or less. Most results indicate that SARS-CoV-2 antibody titers decline slowly (He et al., 2021; L’Huillier et al., 2021; Peluso et al., 2021). A recently published paper analyzing samples collected from early to 12 months after infection indicated that humoral responses to SARS-CoV-2 infection are robust on longer time scales, including responses from mild infection (Laing et al., 2021). Although re-infection by SARS-CoV-2 can occur in patients who previously had symptomatic COVID-19, such cases are infrequent. Recent studies have highlighted a correlation between the presence of SARS-CoV-2 antibodies and a decreased risk of re-infection (Dehgani-Mobaraki et al., 2021; Manisty et al., 2021; Turner et al., 2021).

Analyzing samples collected from patients with mild to severe infection demonstrated that the magnitude of the antibody response to SARS-CoV-2 infection was positively correlated with COVID-19 severity (Pradenas et al., 2021; Trinite et al., 2021; Wu et al., 2021). However, the kinetics of the antibody response in asymptomatic patients is less clear. In this study, hundreds of serum samples were collected from individuals six to twelve months after natural infection with SARS-CoV-2, and the data were collected from August 2020 to March 2021. Serum antibody titers and neutralizing activities were investigated to understand the immune response of patients recovering from asymptomatic SARS-CoV-2 infection. A total of 156 patients with asymptomatic COVID-19 were enrolled in this study. The characteristics of these patients are summarized in Table 1. All of these patients were diagnosed as positive for SARS-CoV-2 in the correctional facility of Shandong Province. All enrolled individuals were asymptomatic after diagnosis.

**TABLE 1:** Characteristics of enrolled patients asymptomatic infected with SARS-CoV-2.

**Characteristic**	**Asymptomatic individuals who tested positive for antibodies at 6-month**
**Sex**	
Male	156/156 (100%)
Female	0/156 (0%)

**Age group, years**	
≤18	0/156 (0%)
18–44	17/156 (10.9%)
44–65	116/156 (74.4%)
≥65	23/156 (14.7%)

Underlying disease*	
No	105/156 (67.3%)
Yes	51/156 (32.7%)

**Medical treatment in the past 1 year**	
No	137/156 (87.8%)
Yes	19/156 (12.2%)

**Self-reported symptom^§^**	
No	156/156 (100%)
Yes	0/156 (0%)

**Medicine use^#^**	
No	148/156 (94.8%)
Yes	8/156 (5.2%)

**Positive for RT-PCR^@^**	
No	0/156 (0%)
Yes	156/156 (100%)

*Underlying diseases included hypertension, respiratory disease, tumors or cancer, diabetes, cardiovascular disease, chronic kidney disease, chronic liver disease, and immunodeficiency disease. ^§^ Including fever or respiratory symptoms, or both. ^#^Including antiviral agent or Antibacterial agent, or both. ^@^RT-PCR test results were positive and diagnosed as a confirmed case.

**FIGURE 1:**
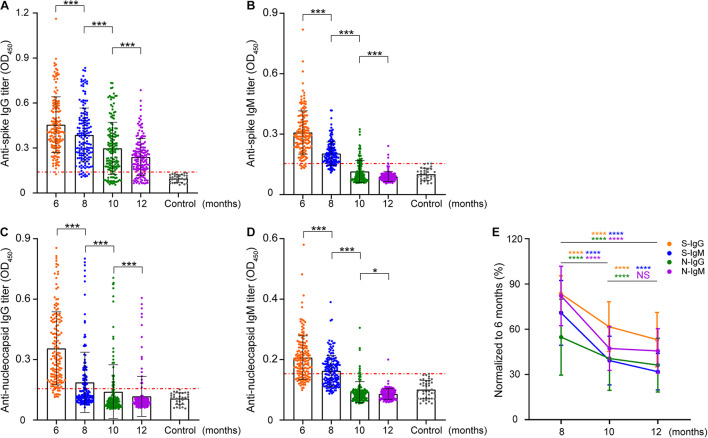
Longitudinal analysis of IgG and IgM against SARS-CoV-2 S/N in patients. The intensity changes of different antibodies were longitudinally compared at a titer of 1:400. **(A–D)** Indicate the longitudinal changes in S-IgG, S-IgM, N-IgG, and N-IgM antibodies. Each data point represents a sample, the vertical line represents the median IQR, the red line marks the cutoff value of enzyme-linked immunosorbent assays (ELISA), and the *y*-axis represents the absorbance at 450 nm when the serum dilution is 1:400. **(E)** Normalization of the OD value of other time points with the OD value of antibodies in June. The *y*-axis represents the proportion of samples in August, October, and December to June for the same patient. The data are shown as the mean ± SEM. The significance is expressed as ns, *P* > 0.05; *P < 0.05; ***P < 0.001; ****P < 0.0001.

The human immune response induced by SARS-CoV-2 can produce multiple proteins that target viral proteins, among which the NP and S proteins have been used as target antigens for serological determination. Therefore, the dynamics of IgG and IgM antibodies against these two proteins were detected first by enzyme-linked immunosorbent assays (ELISA) in this study. The titers of these four antibodies decreased to varying degrees with natural infection (Figure 1). Compared with N-IgG (Figure 1C), S-IgG (Figure 1A) was more stable. S-IgG still existed in some patients 1 year after asymptomatic infection, while N-IgG remained in only a few patients. The titer of IgM (Figures 1B,D) decreased very rapidly and was lower than the baseline 8 months after infection. In general, the rate of decline of IgM was higher than that of IgG (Figure 1E).

**FIGURE 2:**
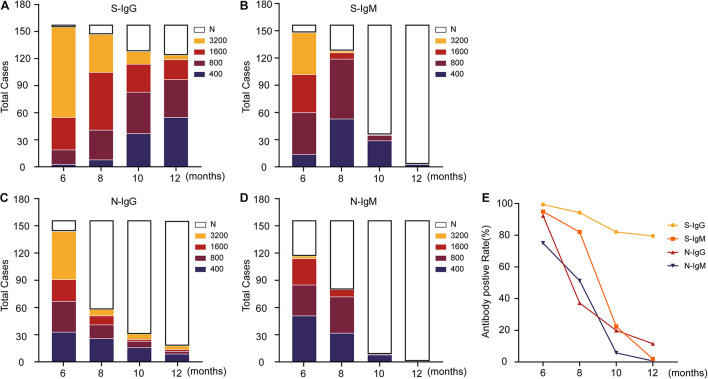
Changing trend of the titers of IgG, IgM and neutralizing antibodies against SARS-CoV-2 S/N in patients. **(A–D)** Indicates the change in antibody titers at different time points, where N indicates that the titers have dropped below the ELISA cutoff and cannot be detected. **(E)** Displays the changing trend of the antibody-positive rate. The data are shown as the mean ± SEM, and the significance is expressed as ns, *P* > 0.05.

To further characterize the positive rate and titer of antibodies, the antibodies were diluted based on 400-fold dilution, with 1:400 defined as a low titer, 1:800 as a medium titer, 1:1,600 as a high titer, and 1:3,200 as an extremely high titer, and then antibodies at different levels were classified. The results showed that the overall positive level of antibodies showed a trend of rapid decline (Figure 2E and Supplementary Figure 1). Compared with the other three antibodies, S-IgG remained stable for a longer time. Approximately 1 year later, S-IgG was still detected in 79% of naturally infected individuals in this study. The IgG response of the N protein decreased to 37% in the 8th month, and only 11.5% of individuals carried N-IgG in the 12th month. The IgM response to the S and N proteins disappeared more rapidly. In the 12th month, antibodies could be detected in less than 2% (S-IgM) and 1% (N-IgM) of individuals.

For the relatively stable S-IgG antibody, the antibody titer also decreased gradually (Figure 2A). Among the four dilutions (400/800/1,600/3,200) used in this study, most S-IgG titers were 3,200 in the 6 months after natural infection and slowly dropped to 400 in the 12 months. The antibody titers of 32 (21%) individuals could not be detected in this experiment. The titer of the N-IgG antibody was reduced significantly 8 months after natural infection (Figure 2C). Antibodies could not be detected in more than half of the patients with natural infection. Most of the patients with detectable antibodies decreased to undetectable levels in the following 4 months. S-IgM (Figure 2B) and N-IgM (Figure 2D) presented a more rapid downward trend and were almost undetectable 12 months after natural infection.

**FIGURE 3:**
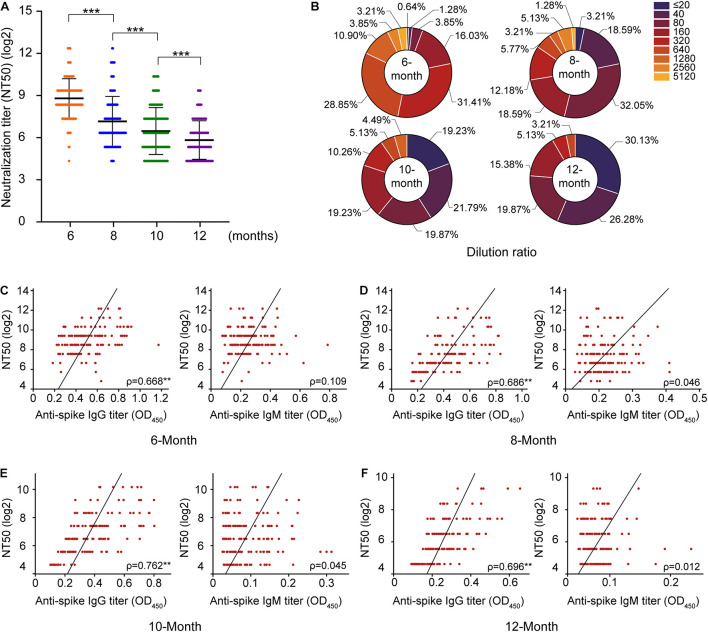
Evaluation of the changes in neutralizing antibody titers in 156 serum samples. According to the classification at different time points after convalescence, **(A)** reflects the change in the neutralization titer of serum from convalescent patients, and the *y*-axis represents the log2 of the neutralization titer. **(B)** Reflects the change in the proportion of the neutralization titer in serum; **(C–F)** indicates the analysis of the correlations of S protein IgG and IgM with neutralizing antibodies, respectively. ***P < 0.001.

Neutralizing antibodies are closely related to the antiviral protection of humoral immunity. The samples were subjected to a pseudovirus neutralization test, and the neutralization titer of the serum was defined with the reciprocal of the serum dilution. This study found that the titer of neutralizing antibodies was stable within 6–12 months after natural asymptomatic infection, but the titer decreased linearly (Figure 3A). The localization of N protein in the viruses and its biological functions indicate that the antibodies against N protein cannot directly neutralize the invasion of SARS-CoV-2, so the correlations of neutralizing antibodies titers with S-IgG and S-IgM levels were analyzed (Figure 3), revealing that S-IgG was more effective than S-IgM in neutralizing SARS-CoV-2 (Figures 3C–F). This effect showed a more significant correlation with S-IgG 12 months after infection, while S-IgM levels were not correlated with neutralizing antibody titers.

Furthermore, the correlations of antibody responses in different periods after natural infection were also analyzed. The significant positive correlations between S/N IgG and S/N IgM were noted, but the correlation becomes weak due to the disappearance of IgM with convalescence (Supplementary Figure 2). Neutralization activity at a serum dilution of 1:160 has been used as a cutoff in a proof-of-concept study showing the efficiency of convalescent plasma therapy. A very high frequency of individuals in our study exhibited strong neutralization activity at 6 months (>=1:160, 98.74%). This proportion decreased to 46.15% at 8 months and 39.11% at 10 months. Only 23.72% of individuals displayed strong neutralization activity at 12 months after natural infection.

From our data, we can conclude that most cases with asymptomatic infection have long-term (12 months) antibody protection, and the level of Ab-protection decreases with time. Previous studies have shown that coronavirus recovered patients can still detect neutralizing antibodies 2 years later (Mo et al., 2006), indicating that detecting antibodies to the SARS-CoV-2 requires a longer period. At the same time, during the 6–12 months of longitudinal testing, we found that the antibody has maintained a relatively low titer level after about 8 months. Unfortunately, The nAb titer minimum level for protection from re-infection is unclear, making it difficult to define the titer of nAb. An experiment on rhesus monkeys shows that the neutralization titer of 83–197 in the pseudovirus neutralization experiment can effectively resist the SARS-CoV-2second attack (Chandrashekar et al., 2020). Therefore, among the asymptomatic cases mentioned in this experiment, it is estimated that nearly half of the patients will have the level of neutralizing antibodies against re-infection with the SARS-CoV-2 in a year.

As we know, antibody levels often determine the clinical outcome of patients, so the key factors that influence antibody levels have been widely discussed by researchers (Siggins et al., 2021; Xiang et al., 2021). The consensus is that people with the severe disease produce high levels of antibodies, meaning they stay in the body longer than those with mild or asymptomatic. Age and gender are also major factors affecting antibody persistence. There is a view that children and elderly patients maintain antibodies for a shorter time after infection than adults, suggesting that children and older adults have weaker long-term memory responses, which may affect the outcome of re-infection (Pierce et al., 2020; Wong et al., 2020). Similarly, gender also affects immune memory. Previous studies have found that gender differences lead to differences in antibody levels, and retrospective analysis has shown that women have higher antibody levels than men, which means they are at lower risk of re-infection (Takahashi et al., 2020; Zeng et al., 2020).

We have failed to conduct in-depth research on the cellular immune memory of asymptomatic infections. According to the work of Gaebler et al. (2021) T and B cell immune memory levels of the patients were stimulated by the SARS-CoV-2 and successfully established. Although immune memory will have a faster response speed than the initial infection of the virus, it still has a certain delay compared with the humoral protection provided by antibodies. A recently published paper showed that individuals in the recovery period of mild and moderate infection Vaccination can produce very long-lasting immunity and protect people from the SARS-CoV-2 variant (Wang et al., 2021). Therefore, from protecting the body from re-infections, we believe that regular vaccination will be required to maintain a high level of antibodies in the body and provide more comprehensive protection for the body.

## Materials and Methods

### Ethics Statement

All experiments were carried out according to procedures approved by the Institute of Microbiology, Chinese Academy of Sciences, and complied with all relevant ethical regulations regarding animal research. The approval certificate of ethics review and consent form to participate in the survey are attached in Supplementary Material.

### Cell Lines

The 293T human embryonic cell line expressing SV40T antigen and the suspension-cultured 293T human embryonic cell line were stored in our laboratory. The 293T cell line stably expressing the hACE2 receptor protein was kindly provided by Dr. Zhendong Zhao (Institute of Pathogen Biology, Chinese Academy of Medical Sciences & Peking Union Medical College). All cells were cultured in DMEM supplemented with 10% FBS (Cat# 26140079, Thermo Fisher Scientific) in 5% CO_2_ at 37°C.

### Sample Collection and Serum Separation

Serum was collected from donors infected and not infected with SARS-CoV-2. Among them, infected samples were collected from 156 convalescent patients with asymptomatic COVID-19 infection in the controlled area of Jining Prison in Shandong Province from August 2020 to April 2021 (Supplementary Figure 3). All the sampled patients were diagnosed and recovered and tested negative by quantitative fluorescence PCR multiple times. Venous blood was collected and allowed to stand for 30 min. After coagulation, the blood was centrifuged at 3,500 × *g* for 10 min, and the serum was aspirated and stored at −80°C for subsequent use. Before use, the viruses were inactivated by heating at 56°C for 1 h. Horseradish peroxidase (HRP)-conjugated antibodies against the human IgG Fc region or human IgM Fc region were used as secondary antibodies to detect serum binding by ELISA.

### Enzyme-Linked Immunosorbent Assays

The recombinant proteins were resuspended in PBS (1 μg/mL), 100 μL of which was collected and added to enzyme plates at 4°C overnight for adsorption. Subsequently, the plates were washed three times with washing buffer (PBS containing 0.02% Tween-20). After blocking with blocking buffer containing 2% BSA and 3% sucrose at 4°C overnight, the plates were blow-dried, vacuumed, and placed at 4°C for subsequent use. The serum samples were diluted to 1:400, 1:800, 1:1,600, and 1:3,200 with dilution buffer (PBS containing 4% BSA), and 50 μL samples were collected and added to enzyme plates for incubation at 37°C for 2 h. After washing four times, secondary antibodies (diluted with dilution buffer at 1:5,000) were added for further incubation at 37°C for 45 min. Afterward, 100 μL of the HRP substrate was added for incubation. Detection was carried out by adding 2 mol/L H_2_SO_4_ to terminate the reaction and reading the absorbance at 450 nm using a microplate reader. Each plate contains a quality control sample. Additionally, the absorbance was read using 30 SARS-CoV-2-negative serum samples at 450 nm to calculate the cutoff with the following formula (Frey et al., 1998; Lardeux et al., 2016) to determine the final positive threshold for ELISA.


$$\text{Cutoff}=\overset{\_\,\_}{X}+S\,D\,t\,\sqrt{1+\left(1\,/\,n\right)}$$

(*T* = 1.727), the cutoff value for S-IgG was 0.1397, S-IgM was 0.1530, N-IgG was 0.1550, and N-IgM was 0.1522. The serum sample is considered positive when the OD is above the cutoff value.

### Virus Neutralization Assay

293T-hACE2 cells were infected with SARS-CoV-2 pseudoviruses expressing luciferase in culture dishes containing serum from convalescent patients. In brief, 293T cells were cotransfected with the three-plasmid lentivirus-packaging system (psPAX2, pLenti-GFP and coding SARS-CoV-2S) and liposomes at 1:2, with 100 μg of cells transfected per 15 cm dish. After transfection for 48 h, the supernatant was collected, centrifuged at 4,000 × *g* for 10 min to remove cell debris, further filtered using a 0.45-μm mesh, and finally stored at −80°C for subsequent use. Twenty-four hours before the neutralization assay, 293T-hACE2 cells were seeded into 96-well plates at 3 × 10^4^/well. Serum samples were diluted from 1:10 to 1:5,120, and 50 μL was collected and mixed with isovolumic viruses for incubation at 37°C for 1 h, which was then added to target cells for continuous incubation at 37°C for 48 h. Detection was performed by adding 100 μL of luciferase substrate and transferring it to 96-well plates in the dark for reading using a microplate reader. The neutralizing antibody titer of each serum sample was expressed as the 50% neutralizing titer (NT50), which was defined as the reciprocal of the highest serum dilution with a 50% neutralization rate.

### Statistical Analysis

The significance of the results was tested using GraphPad Prism (version 8.0.2). Statistical tests (parametric or non-parametric) with *P* < 0.05 were considered significant. The significance is expressed as ns, *P* > 0.05; ^∗^*P* < 0.05; ^∗∗^*P* < 0.01; ^∗∗∗^*P* < 0.001. The Spearman rank correlation of the data was tested using IBM SPSS statistics (version 26).

## Data Availability Statement

The original contributions presented in the study are included in the article/Supplementary Material, further inquiries can be directed to the corresponding authors.

## Ethics Statement

The studies involving human participants were reviewed and approved by the Institute of Microbiology, Chinese Academy of Sciences. The patients/participants provided their written informed consent to participate in this study.

## Author Contributions

JL initiated and coordinated the project. JL and WL designed the experiments. XT prepared the pseudotype SARS-CoV-2 virus, evaluated the neutralizing potency using pseudovirus. XT, HZ, and WJ participated in the sampling. JL and XT prepared the manuscript and completed its revision and performed the data analyses. WL suggested many of the experiments in this study. All authors read and approved the final manuscript.

## Conflict of Interest

The authors declare that the research was conducted in the absence of any commercial or financial relationships that could be construed as a potential conflict of interest.

## Publisher’s Note

All claims expressed in this article are solely those of the authors and do not necessarily represent those of their affiliated organizations, or those of the publisher, the editors and the reviewers. Any product that may be evaluated in this article, or claim that may be made by its manufacturer, is not guaranteed or endorsed by the publisher.

## Supplementary Material

Supplementary Figure 1 The change in antibody titers with dilution. The dotted line represents the cutoff for ELISA, the horizontal coordinate represents three different dilutions, and the longitudinal coordinate represents the change in OD value with dilution.

Supplementary Figure 2 Correlations of antibody responses in different periods after natural infection. S-IgG, S-IgM, N-IgG, and N-IgM titers are strongly correlated, but the correlation becomes weak due to the disappearance of IgM with convalescence. Orange indicates a tendency toward a higher correlation, purple indicates a weak correlation, and ^∗^ represents a significant correlation.

Supplementary Figure 3 The serum samples were collected from the flow chart of patients asymptomatic infected with COVID-19 from August 2020 to March 2021.

## References

[B1] Anand S. P. Prevost J. Nayrac M. Beaudoin-Bussieres G. Benlarbi M. Gasser R. (2021). Longitudinal analysis of humoral immunity against SARS-CoV-2 Spike in convalescent individuals up to eight months post-symptom onset. *Cell Rep. Med.* 2:100290. 10.1016/j.xcrm.2021.100290 PMC8097665 33969322

[B2] Chandrashekar A. Liu J. Martinot A. J. McMahan K. Mercado N. B. Peter L. (2020). SARS-CoV-2 infection protects against rechallenge in rhesus macaques. *Science* 369 812–817. 32434946 10.1126/science.abc4776 PMC7243369

[B3] Cohen K. W. Linderman S. L. Moodie Z. Czartoski J. Lai L. Mantus G. (2021). Longitudinal analysis shows durable and broad immune memory after SARS-CoV-2 infection with persisting antibody responses and memory B and T cells. *medRxiv* [Preprint]. 10.1016/j.xcrm.2021.100354 34250512 PMC8253687

[B4] Dehgani-Mobaraki P. Zaidi A. K. Porreca A. Floridi A. Floridi E. (2021). Neutralizing antibody responses 10 months after mild and moderately-severe SARS-CoV-2 infection. *medRxiv* [Preprint]. 10.1101/2021.02.22.21252225

[B5] Figueiredo-Campos P. Blankenhaus B. Mota C. Gomes A. Serrano M. Ariotti S. (2020). Seroprevalence of anti-SARS-CoV-2 antibodies in COVID-19 patients and healthy volunteers up to 6 months post disease onset. *Eur. J. Immunol.* 50 2025–2040. 10.1002/eji.202048970 33084029 PMC7756220

[B6] Frey A. Di Canzio J. Zurakowski D. (1998). A statistically defined endpoint titer determination method for immunoassays. *J. Immunol. Methods* 221 35–41. 10.1016/s0022-1759(98)00170-7 9894896

[B7] Gaebler C. Wang Z. Lorenzi J. C. Muecksch F. Finkin S. Tokuyama M. (2021). Evolution of antibody immunity to SARS-CoV-2. *Nature* 591 639–644. 33461210 10.1038/s41586-021-03207-w PMC8221082

[B8] Galipeau Y. Greig M. Liu G. Driedger M. Langlois M. A. (2020). Humoral responses and serological assays in SARS-CoV-2 infections. *Front. Immunol.* 11:610688. 10.3389/fimmu.2020.610688 33391281 PMC7775512

[B9] Garcia-Beltran W. F. Lam E. C. Astudillo M. G. Yang D. Miller T. E. Feldman J. (2021). COVID-19-neutralizing antibodies predict disease severity and survival. *Cell* 184 476.e11–488.e11. 10.1016/j.cell.2020.12.015 33412089 PMC7837114

[B10] Garrido C. Hurst J. H. Lorang C. G. Aquino J. N. Rodriguez J. Pfeiffer T. S. (2021). Asymptomatic or mild symptomatic SARS-CoV-2 infection elicits durable neutralizing antibody responses in children and adolescents. *medRxiv* [Preprint]. 10.1101/2021.04.17.21255663 34228642 PMC8492306

[B11] Gudbjartsson D. F. Norddahl G. L. Melsted P. Gunnarsdottir K. Holm H. Eythorsson E. (2020). Humoral immune response to SARS-CoV-2 in Iceland. *N. Engl. J. Med.* 383 1724–1734. 32871063 10.1056/NEJMoa2026116 PMC7494247

[B12] Hashem A. M. Algaissi A. Almahboub S. A. Alfaleh M. A. Abujamel T. S. Alamri S. S. (2020). Early humoral response correlates with disease severity and outcomes in COVID-19 patients. *Viruses* 12:1390. 10.3390/v12121390 33291713 PMC7761967

[B13] He Z. Ren L. Yang J. Guo L. Feng L. Ma C. (2021). Seroprevalence and humoral immune durability of anti-SARS-CoV-2 antibodies in Wuhan, China: a longitudinal, population-level, cross-sectional study. *Lancet* 397 1075–1084. 10.1016/S0140-6736(21)00238-5 33743869 PMC7972311

[B14] Ibarrondo F. J. Fulcher J. A. Goodman-Meza D. Elliott J. Hofmann C. Hausner M. A. (2020). Rapid decay of Anti-SARS-CoV-2 antibodies in persons with mild Covid-19. *N. Engl. J. Med.* 383 1085–1087. 10.1056/nejmc2025179 32706954 PMC7397184

[B15] Laing E. D. Epsi N. J. Richard S. A. Samuels E. C. Wang W. Vassell R. (2021). SARS-CoV-2 antibodies remain detectable 12 months after infection and antibody magnitude is associated with age and COVID-19 severity. *medRxiv* [Preprint]. 10.1101/2021.04.27.21256207

[B16] Lardeux F. Torrico G. Aliaga C. (2016). Calculation of the ELISA’s cutoff based on the change-point analysis method for detection of Trypanosoma cruzi infection in Bolivian dogs in the absence of controls. *Mem. Inst. Oswaldo Cruz* 111 501–504. 10.1590/0074-02760160119 27384081 PMC4981115

[B17] L’Huillier A. G. Meyer B. Andrey D. O. Arm-Vernez I. Baggio S. Didierlaurent A. (2021). Antibody persistence in the first 6 months following SARS-CoV-2 infection among hospital workers: a prospective longitudinal study. *Clin. Microbiol. Infect.* 27 784.e1–784.e8. 10.1016/j.cmi.2021.01.005 PMC7816882 33482352

[B18] Li J. Liu W. (2020). Puzzle of highly pathogenic human coronaviruses (2019-nCoV). *Protein Cell* 11 235–238. 32088858 10.1007/s13238-020-00693-y PMC7093363

[B19] Manisty C. Treibel T. A. Jensen M. Semper A. Joy G. Gupta R. K. (2021). Time series analysis and mechanistic modelling of heterogeneity and sero-reversion in antibody responses to mild SARS-CoV-2 infection. *EBioMedicine* 65:103259. 10.1016/j.ebiom.2021.103259 33662833 PMC7920816

[B20] Mo H. Zeng G. Ren X. Li H. Ke C. Tan Y. (2006). Longitudinal profile of antibodies against SARS-coronavirus in SARS patients and their clinical significance. *Respirology* 11 49–53. 10.1111/j.1440-1843.2006.00783.x 16423201 PMC7192223

[B21] Peluso M. J. Deitchman A. N. Torres L. Iyer N. S. Nixon C. C. Munter S. E. (2021). Long-term SARS-CoV-2-specific immune and inflammatory responses across a clinically diverse cohort of individuals recovering from COVID-19. *medRxiv* [Preprint]. 10.1101/2021.02.26.21252308 34358460 PMC8342976

[B22] Pierce C. A. Preston-Hurlburt P. Dai Y. Aschner C. B. Cheshenko N. Galen B. (2020). Immune responses to SARS-CoV-2 infection in hospitalized pediatric and adult patients. *Sci. Transl. Med.* 12:eabd5487. 10.1126/scitranslmed.abd5487 PMC7658796 32958614

[B23] Pradenas E. Trinité B. Urrea V. Marfil S. Ávila-Nieto C. de la Concepción M. L. R. (2021). Stable neutralizing antibody levels 6 months after mild and severe COVID-19 episodes. *Med* 2 313.e4–320.e4. 10.1016/j.medj.2021.01.005 33554155 PMC7847406

[B24] Siggins M. K. Thwaites R. S. Openshaw P. J. (2021). Durability of immunity to SARS-CoV-2 and other respiratory viruses. *Trends Microbiol.* 29 648–662. 10.1016/j.tim.2021.03.016 33896688 PMC8026254

[B25] Takahashi T. Ellingson M. K. Wong P. Israelow B. Lucas C. Klein J. (2020). Sex differences in immune responses that underlie COVID-19 disease outcomes. *Nature* 588 315–320. 32846427 10.1038/s41586-020-2700-3 PMC7725931

[B26] Trinite B. Tarres-Freixas F. Rodon J. Pradenas E. Urrea V. Marfil S. (2021). SARS-CoV-2 infection elicits a rapid neutralizing antibody response that correlates with disease severity. *Sci. Rep.* 11:2608. 10.1038/s41598-021-81862-9 PMC7843981 33510275

[B27] Turner J. S. Kim W. Kalaidina E. Goss C. W. Rauseo A. M. Schmitz A. J. (2021). SARS-CoV-2 infection induces long-lived bone marrow plasma cells in humans. *Nature* 595 421–425. 10.1038/s41586-021-03647-4 34030176

[B28] Wang Y. Zhang L. Sang L. Ye F. Ruan S. Zhong B. (2020). Kinetics of viral load and antibody response in relation to COVID-19 severity. *J. Clin. Invest.* 130 5235–5244. 32634129 10.1172/JCI138759 PMC7524490

[B29] Wang Z. Muecksch F. Schaefer-Babajew D. Finkin S. Viant C. Gaebler C. (2021). Naturally enhanced neutralizing breadth against SARS-CoV-2 one year after infection. *Nature* 595 426–431. 34126625 10.1038/s41586-021-03696-9 PMC8277577

[B30] Wong L. S. Y. Loo E. X. L. Kang A. Y. H. Lau H. X. Tambyah P. A. Tham E. H. (2020). Age-related differences in immunological responses to SARS-CoV-2. *J. Allergy Clin. Immunol.* 8 3251–3258. 10.1016/j.jaip.2020.08.026 32861856 PMC7450283

[B31] World Health Organization [WHO] (2020). *WHO Coronavirus (COVID-19) Dashboard.* Avaliable online at: https://covid19.who.int/ (accessed May 31, 2021).

[B32] Wu J. Liang B. Chen C. Wang H. Fang Y. Shen S. (2021). SARS-CoV-2 infection induces sustained humoral immune responses in convalescent patients following symptomatic COVID-19. *Nat. Commun.* 12:1813. 10.1038/s41467-021-22034-1 33753738 PMC7985370

[B33] Xiang T. Liang B. Fang Y. Lu S. Li S. Wang H. (2021). Declining levels of neutralizing antibodies against SARS-CoV-2 in Convalescent COVID-19 patients one year post symptom onset. *Front. Immunol.* 12:2327. 10.3389/fimmu.2021.708523 PMC8242354 34220870

[B34] Zeng F. Dai C. Cai P. Wang J. Xu L. Li J. (2020). A comparison study of SARS-CoV-2 IgG antibody between male and female COVID-19 patients: a possible reason underlying different outcome between sex. *J. Med. Virol.* 92 2050–2054. 32383183 10.1002/jmv.25989 PMC7267228

[B35] Zheng Y. Zhang Q. Ali A. Li K. Shao N. Zhou X. (2021). Sustainability of SARS-CoV-2 induced humoral immune responses in COVID-19 patients from hospitalization to convalescence over six months. *Virol. Sin.* 1–10. 33661489 10.1007/s12250-021-00360-4 PMC7931792

