# Supplementary figures and images for: Persistence of the SARS-CoV-2 Antibody Response in Asymptomatic Patients in Correctional Facilities
Source: Front Microbiol. 2021 Nov 10;12:789374. doi: 10.3389/fmicb.2021.789374 (PMC8631518; doi:10.3389/fmicb.2021.789374)

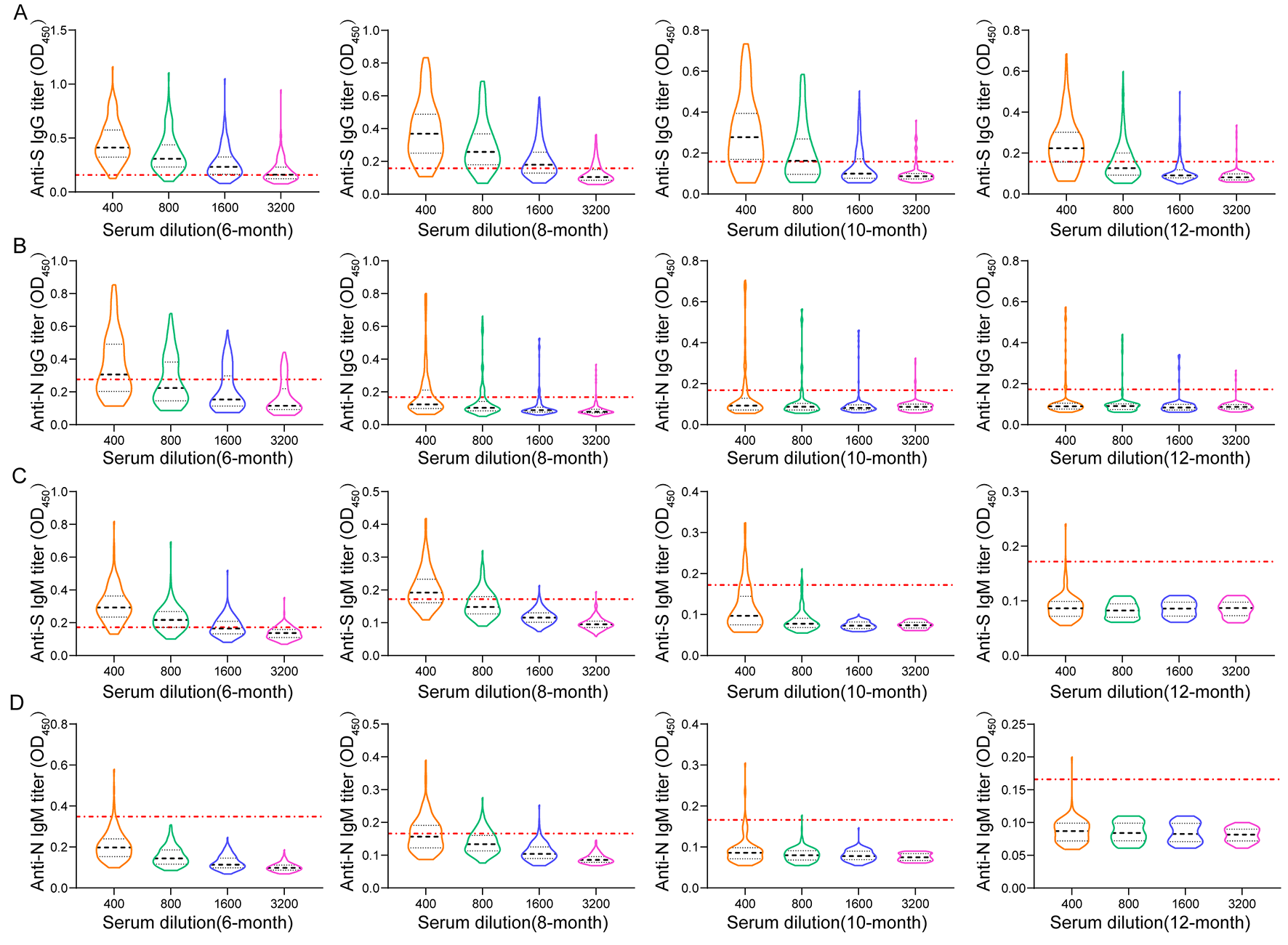

Supplement: Supplementary Figure 1 — The change in antibody titers with dilution. The dotted line represents the cutoff for ELISA, the horizontal coordinate represents three different dilutions, and the longitudinal coordinate represents the change in OD value with dilution. [file Image_1.TIF]

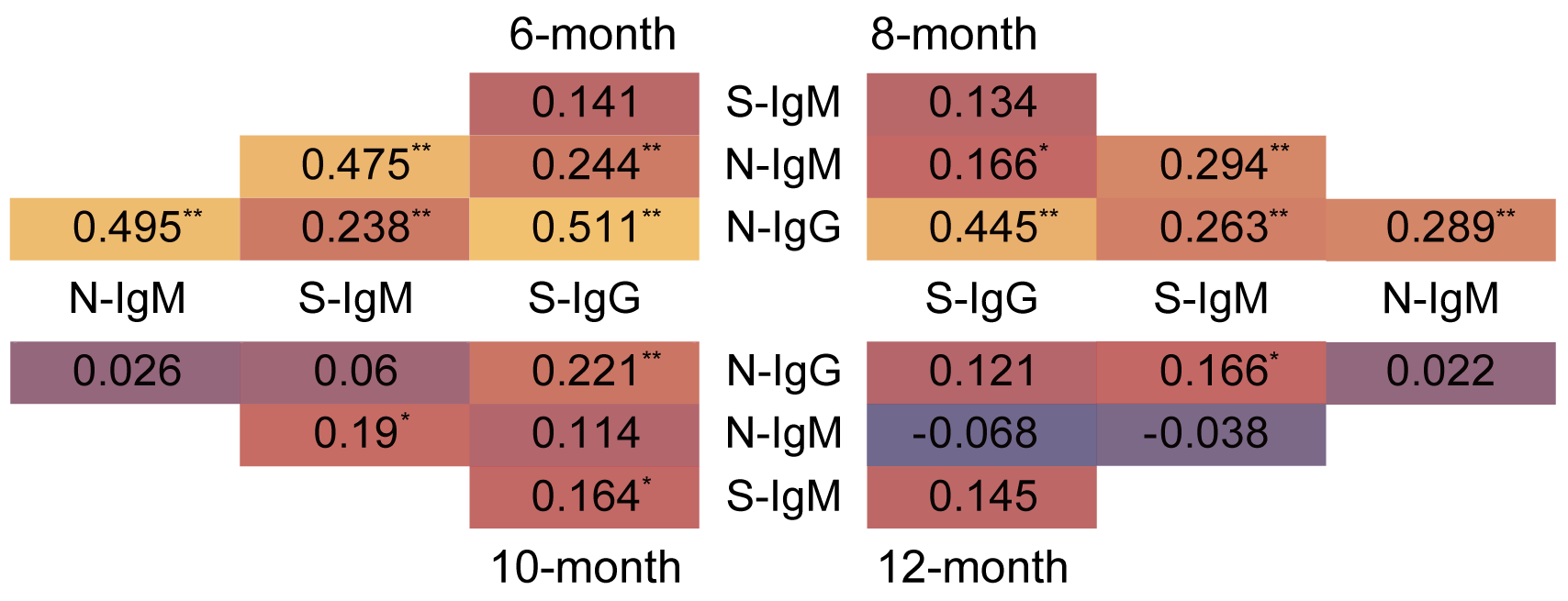

Supplement: Supplementary Figure 2 — Correlations of antibody responses in different periods after natural infection. S-IgG, S-IgM, N-IgG, and N-IgM titers are strongly correlated, but the correlation becomes weak due to the disappearance of IgM with convalescence. Orange indicates a tendency toward a higher correlation, purple indicates a weak correlation, and ∗ represents a significant correlation. [file Image_2.TIF]

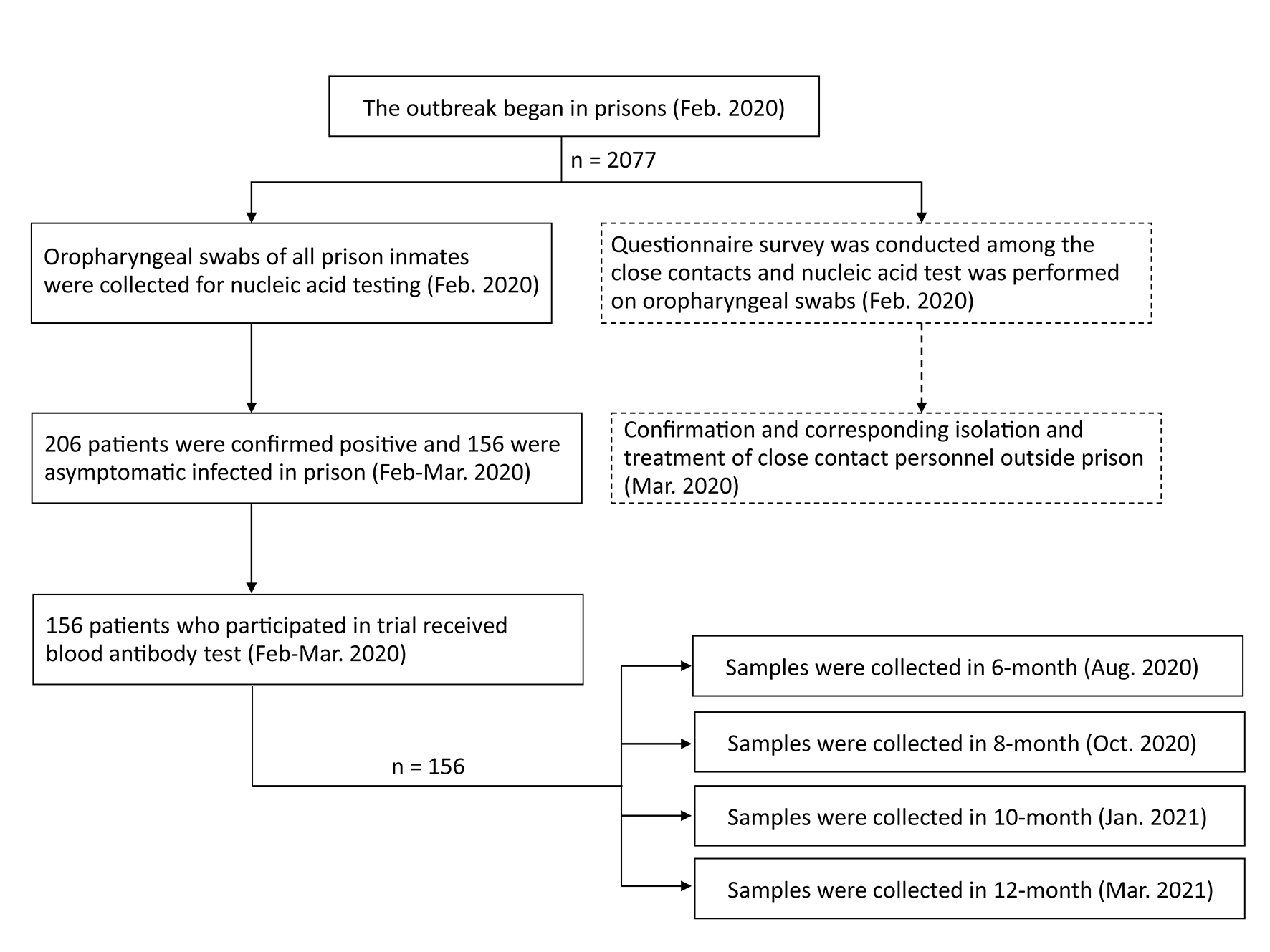

Supplement: Supplementary Figure 3 — The serum samples were collected from the flow chart of patients asymptomatic infected with COVID-19 from August 2020 to March 2021. [file Image_3.TIF]
